# Emergence of Colistin-Resistant *Acinetobacter junii* in China

**DOI:** 10.3390/antibiotics11121693

**Published:** 2022-11-24

**Authors:** Zhiren Wang, Xuanyang Fan, Shuyi Wang, Shuguang Li, Yue Gao, Hui Wang, Henan Li

**Affiliations:** Department of Clinical Laboratory, Peking University People’s Hospital, Beijing 100044, China

**Keywords:** *Acinetobacter junii*, colistin resistance, PmrABC, *lpxA*, *lpxD*

## Abstract

The increasing number of multidrug-resistant Gram-negative bacteria presents a serious threat to global health. However, colistin-resistant *Acinetobacter junii* has rarely been reported. We identified a colistin-resistant *A. junii* clinical isolate, AJ6079, in blood. The colony of AJ6079 presented a dry phenotype, and it was difficult to form a bacterial suspension, whilst transmission electron microscopy revealed that AJ6079 possessed a thick outer membrane. The phenotypic and genomic comparisons were conducted with one colistin-susceptible *A. junii*, which had the same antibiotic susceptibility profile except for colistin, and had the same KL25 capsule biosynthesis locus. The AJ6079 exhibited a slower growth rate, indicating that colistin-resistant *A. junii* possesses a higher fitness cost. The genome of AJ6079 had a G+C content of 38.7% and contained one 3,362,966 bp circular chromosome with no plasmid or mobile colistin resistance (*mcr*) gene. Comparative genomic analysis revealed that the AJ6079 contained several previously unreported point mutations in colistin-resistance-related genes involving amino acid substitutions in PmrB (N5K, G147C), LpxA (I107F, H131Y), and LpxD (F20I, K263R), which might be correlated with colistin resistance in *A. junii*. Further research is needed for verification as the genetic background was not exactly the same between the two isolates.

## 1. Introduction

*Acinetobacter* is a Gram-negative bacillus that is widely distributed in the environment, mainly in water and soil, and can readily survive in humid environments. *Acinetobacter* can colonize the skin, wounds, respiratory tract, gastrointestinal tract, and oral mucosa of humans. Although there are more than 50 species within the diverse *Acinetobacter* genus, the most common species that cause infections include *Acinetobacter baumannii*, *Acinetobacter haemolyticus*, *Acinetobacter junii*, *Acinetobacter nosocomialis*, and *Acinetobacter lwoffii* [1]. In recent years, *Acinetobacter* infections have been on the rise, with an increase in antibiotic resistance that has become a worldwide public health problem. Most clinical reports are related to multidrug-resistant *A. baumannii*, and the elevated rates of colistin resistance exert pressure on clinical management and strongly impact patient outcomes. However, in addition to *A. baumannii*, other members of the genus, such as *A. junii*, have become important in clinical settings, particularly concerning bacteremia in newborns, pediatric oncology patients, and adult immunocompromised patients [2].

With the continuous clinical use of colistin, the emergence of colistin-resistant isolates has increased since the first report of colistin-resistant *Acinetobacter spp.* in the Czech Republic in 1999 [3]. According to the prevalence of antibiotic-resistant *A. baumannii* reported by WHO regional offices from 1999 to 2017, Lebanon (17.5%) and China (12%) had the highest colistin resistance rates among 41 countries from different regions of the world [4]. Colistin resistance mechanisms in *A. baumannii* have been widely studied and mainly involve modifications to the lipopolysaccharide (LPS) structure and charge. There are two main LPS modifications investing colistin resistance, including the addition of 4-amino-4-deoxy-l-arabinose and phosphoethanolamine (PetN) to the lipid A [5]. In the absence of genes that synthesize 4-amino-4-deoxy-l-arabinose, there exists only one modification pathway for PetN in *A. baumannii*, which relies on the PetN transferase PmrC and reaction regulatory proteins sensor kinase PmrB and transcriptional regulator PmrA in a two-component system [6,7]. Mutations in *pmrC* and the two-component system genes *pmrAB* can change the membrane potential, leading to LPS modification. Mutations involving the LPS biosynthetic genes *lpxA* (UDP-N-acetylglucosamine O-acyltransferase encoding gene), *lpxC* (UDP-3-O-(R-3-hydroxymyristoyl)-N-acetylglucosamine deacetylase encoding gene), and *lpxD* (UDP-3-O-acyl-glucosamine N-acyltransferase encoding gene) can hinder lipid A biosynthesis, leading to the loss of the colistin target [6]. The insertion sequence (IS) IS*Aba1* can mediate the overexpression of lipid A phosphoethanolamine transferase encoding gene *eptA*, which possesses 93% homology to *pmrC*, resulting in colistin resistance [5]. The emergence of the plasmid-borne mobile colistin resistance (*mcr*) gene has accelerated the dissemination of colistin resistance [8]. Ten *mcr* genes have been identified, among which, *A. baumannii* may carry *mcr*-1 and *mcr*-4.3, all of which are associated with high-level drug resistance [9]. Moreover, efflux pump overexpression also plays an important role in the development of colistin resistance [6]. However, colistin-resistant *A. junii* strains have been rarely reported. Here, we identified a colistin-resistant clinical *A. junii* isolate and explored its genotypic and phenotypic profiles.

## 2. Results and Discussion

### 2.1. Phenotypic Characterization of AJ6079

**Figure 1:**
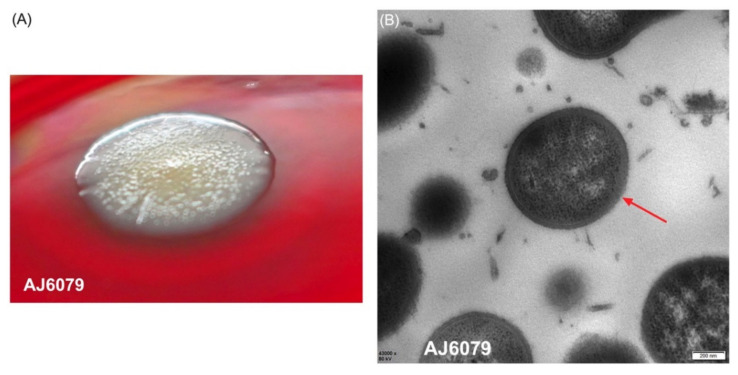
Morphology of AJ6079. (**A**) Colonies of colistin-resistant *A. junii* AJ6079 on a Columbia blood agar plate. (**B**) Microscopic morphology of AJ6079 under TEM. Scale bars = 200 nm. Arrow indicates the bacterial outer membrane.

Colistin-resistant *A. junii* AJ6079 is a clinical isolate collected from the blood of a patient with bloodstream infection. The isolates inoculated on Columbia blood agar plates were dry and flat with a rough surface, enabling them to agglomerate and become difficult to separate (Figure 1A). The bacterial clumps were suspended in 0.9% saline solution and remained difficult to separate from each other. The dry and wrinkled colony phenotype may lead to the failure of bacterial dispersion and suspension. A specific morphotype named red, dry, and rough (RDAR) has been reported in *Salmonella enterica*, which exhibits multicellular behavior resistant to various environmental stresses, including desiccation and nutrient deprivation, and allows for biofilm formation [10]. RDAR colonies resulted from the expression of curli, cellulose, and other polysaccharides. The similar characteristics of AJ6079 indicated that these particular morphologies might be correlated to fimbriae and polysaccharide synthesis, which requires further investigation. 

We investigated the microscopic morphology of AJ6079 cells using transmission electron microscopy (TEM). AJ6079 had a rounded oval shape with a thick bacterial outer membrane (OM) that was visible under an electron microscope (Figure 1B). The OM of Gram-negative bacteria is a unique asymmetric lipid bilayer composed of phospholipids in the inner leaflet and LPS in the outer leaflet, constituting an efficient selective permeation barrier to protect the cell from noxious compounds, while also making Gram-negative bacteria more resistant to antibiotics than Gram-positive bacteria [11]. A recent study showed that *mcr-1*-mediated colistin resistance in *E. coli* is due to LPS modification of the cytoplasmic membrane instead of OM, demonstrating that colistin exerts its bactericidal effect by targeting LPS in the cytoplasmic membrane [12]. Colistin can bind to LPS on bacterial surfaces and disintegrate OM [8]. Colistin might traverse the OM to reach the cytoplasmic membrane, which is required for bacterial lysis and killing via a process of “self-directed uptake”, when the LPS monolayer is damaged. Therefore, a thick OM provides greater resistance to colistin, permeabilizes the bacterial membrane, and might slow the rate at which colistin exerts bactericidal effects.

We conducted antibiotic susceptibility tests to identify the resistance profile of AJ6079. The results revealed that AJ6079 was only resistant to colistin (minimum inhibitory concentration (MIC) = 4 mg/L) but susceptible to other antibiotics, including carbapenems, aminoglycosides, quinolones, and tigecycline (Table 1). Ocular colistin-resistant Gram-negative bacilli are reported to remain susceptible to carbapenems (83.3%), aminoglycosides (58.3%), and fluoroquinolones (62.5%) [13]. In a study of bacteremia caused by *A. junii*, 35% of isolates were resistant to colistin, whereas all isolates were susceptible to carbapenem and levofloxacin [2]. Thus, we need to strengthen the surveillance and management of colistin usage and the distribution and dissemination of colistin resistance genes to control the emergence of colistin-resistant bacteria.

**Figure 2:**
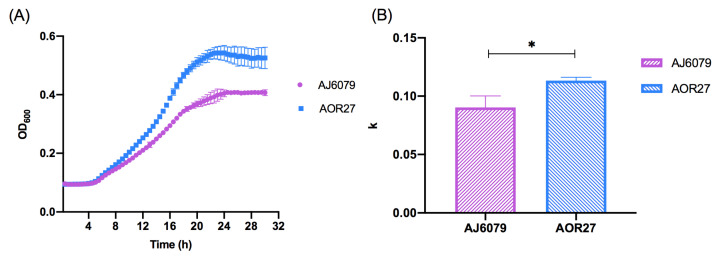
Phenotypic discrepancies between colistin-resistant and -susceptible *Acinetobacter junii*. (**A**) Growth curves of AJ6079 and AOR27. AJ6079, colistin-resistant *A. junii*; AOR27, colistin-susceptible *A. junii*. (**B**) Growth analysis of AJ6079 and AOR27. The ordinate represents the corrected *k* value of the nonlinear fitting logistic growth and data were analyzed using the corrected Welch’s *t*-test. An asterisk (*) indicates a statistically significant difference (*P* < 0.05).

To investigate the phenotypic impact of colistin resistance, we compared the growth rate of AJ6079 to that of colistin-susceptible *A. junii* AOR27 (Table 1). Compared with AJ6079, AOR27 had the same antibiotics susceptibility profile except for colistin. The selected colistin-susceptible isolate AOR27 had the same KL25 capsule biosynthesis locus as AJ6079, but the genetic background was not exactly the same between the two isolates. Growth curve measurements showed that AJ6079 grew much slower (*P* < 0.05, Figure 2), indicating a higher fitness cost. Most studies have found the same fitness decrease in colistin-resistant *A. baumannii*, which is related to LPS loss or modifications [6]. Resistance mutations may have some impacts on the indispensable functions of isolates and modulate their adaptation.

### 2.2. Genome Analysis of AJ6079

Whole genome sequencing analysis showed that the genome of AJ6079 contained one 3,362,966 bp circular chromosome with a G+C content of 38.7% and no plasmid. A total of 3181 genes were annotated using the Prokka software. The coding sequence coverage rate over the whole genome was 87.4%, with an average depth of 793.33. The sequence was identified as *A.junii* at the species level by the ribosomal multilocus sequence typing. We analyzed the potential resistance genes using ResFinder, and no known resistance genes were detected. This was consistent with the antimicrobial susceptibility results, indicating that colistin resistance might be relevant to chromosome mutations. To further analyze colistin resistance-associated genomic variation, we compared colistin-susceptible *A. junii* AOR27 with AJ6079. The core genes were found to account for 68.99% (2534/3673) of the total genes through core genome comparison with Roary, and accounted for 83.32% (2727/3273) through the total annotated genes with sequence-based comparison using SEED viewer. Meanwhile, AOR27 was characterized by the same KL25 capsule biosynthesis locus as AJ6079. We then extracted the known colistin-resistance-related genes *pmrA*, *pmrB*, *pmrC*, *lpxA*, *lpxC*, *lpxD*, and *mcr* from AJ6079 and AOR27 and performed multiple sequence alignment using BLAST. A genome comparison investigation found that there were mutations in *pmrB*, *pmrC*, *lpxA*, and *lpxD* in AJ6079, whereas no *mcr* genes were found. 

**Figure 3:**
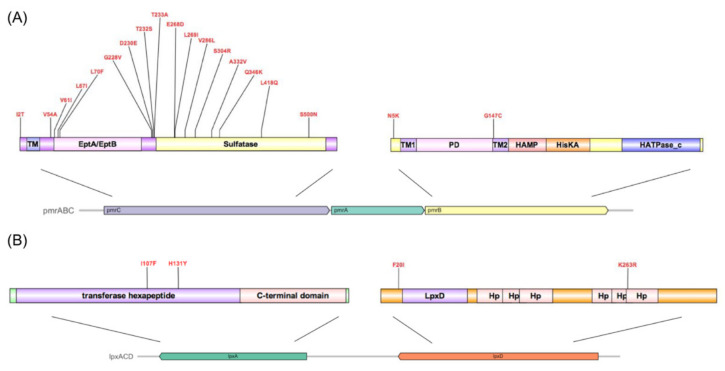
Genotypic discrepancies in colistin-resistance-related genes between colistin-resistant and -susceptible *Acinetobacter junii.* (**A**) Amino acid substitution sites in PmrABC. The arrows depict the cluster constituent genes with the names on them. Different predicted functional domains are colored and annotated, respectively. TM, transmembrane region; PD, Periplasmic domain. (**B**) Amino acid substitution sites in LpxAD. The arrows depict the cluster constituent genes with the names on them. Different functional domains are colored and annotated, respectively. Hp, hexapeptide.

The amino acid mutation sites of AJ6079 in the two-component signal transduction system histidine kinase PmrB were N5K and G147C (Figure 3A). Many amino acid substitutions or short amino acid deletions have been identified in PmrAB among colistin-resistant isolates [6]. All mutations in the response regulator PmrA are located in the phosphate receiver domain, whereas histidine kinase PmrB is reported to be a more common site for gain-of-function mutations, which occurred in four of the six predicted domains. Mutations in *pmrB* have been reported to carry a fitness cost, which might be the reason for the slow growth and high fitness cost of AJ6079 [14]. The mutation site Gly147 in PmrB localized to the second transmembrane domain, which participates in sensing external stimuli and protein signaling [15]. Substitution in the transmembrane domain may cause domain conformation loss and promote the phosphorylation of PmrB. Various PmrC mutation sites in AJ6079 (I2T, V54A, V61I, L67I, L70F, G228V, D230E, T232S, T233A, E268D, L269I, V286L, S304R, A332V, Q346K, L418Q, and S500N) may subtly affect colistin resistance. Many studies have also found that *pmrC* in colistin-resistant isolates contains various missense mutations, whereas isolates still harbor mutations in additional colistin-resistance-related genes, which is consistent with our findings [16,17]. The variability of *pmrC* indicates that it might play a minor role in colistin resistance, and its contribution to resistance has not yet been deciphered, which requires further functional studies. It has been reported that amino acid substitutions (T614A), frameshift mutations (frameshift after S153), and IS disruptions in UDP-N-acetylglucosamine O-acyltransferase LpxA cause the loss of LPS, reducing colistin susceptibility [18,19]. Recent research has shown that mutations in UDP-3-O-glucosamine N-acyltransferase LpxD are also associated with colistin resistance [20]. However, the I107F and H131Y mutations in LpxA and the F20I and K263R mutations in LpxD found in AJ6079 were not among the reported mutations (Figure 3B). The crystal structures and binding domains of LpxA and LpxD in *A. baumannii* have been thoroughly investigated. The LpxA amino acid substitutions sites Ile107 and His131 were inside the UDP-binding Pocket, while the His131 was located near the crucial residue Ile133, which might affect its UDP combination and interfere with its function [21,22]. The LpxD amino acid substitution sites Phe20 and Lys263 are located in the uridine-binding and lipid-binding domains, respectively, leading to a possible impact on enzyme function and polymerization [20].

The compared investigation of virulence-related genes between AJ6079 and colistin-susceptible *A. junii* isolate AOR27 revealed that AJ6079 possessed additional capsule-related genes (glycosyltransferase encoding gene ACICU_RS00470 and sugar transferase encoding gene ACICU_RS00475) and the type II secretion system effector metalloendopeptidase encoding gene *cpaA*, while the β-1,6-poly-N-acetyl-D-glucosamine (PNAG)-related PNAG synthase encoding gene *pgaC* was complete in AOR27 and partial in AJ6079.

This study has some limitations. First, the isogenic relationship between colistin-resistance *A.junii* AJ6079 and colistin-susceptible *A.junii* AOR27 was not solid enough to support the causal relationship between phenotype changes and colistin resistant-related mutations. Further constructions of the point mutations and investigation under the isogenic environment are needed. The results of electron microscopy and structural analysis suggested that colistin-resistance-related mutations may be related to the function of the OM and enzyme. Proteomic research should be conducted in the future. Second, the sample size was relatively small for the presumption of a relationship between mutations, resistance, and fitness changes. The number of *A. junii* isolates that include both antibiotic susceptibility and genome sequence information in public databases is rare. Additionally, the colistin susceptibility level of AJ6079 was close to the proposed resistance breakpoints and the phenome has been characterized as heteroresistance subpopulations in a previous study [23]. The method we used to determine MICs could not distinguish whether the strain was heteroresistant to colistin. Our study provided clues for further elucidating the mechanism of colistin resistance in *A. junii*. More antibiotic susceptibility and genomic data for *A. junii* are required to determine whether the findings described here can be generalized. 

## 3. Materials and Methods

### 3.1. Bacterial Strains and Antimicrobial Susceptibility Test

Colistin-resistant *A. junii* AJ6079 was screened from 1590 representative isolates collected from 11 hospitals in China. The MICs of 10 common antibiotics (imipenem, meropenem, ceftazidime, levofloxacin, minocycline, tigecycline, eravacycline, colistin, amikacin, and trimethoprim/sulfamethoxazole) were determined using broth microdilution assays. The experimental results were interpreted according to the Clinical and Laboratory Standards Institute performance standards for antimicrobial susceptibility M100-S29, whereas tigecycline was interpreted according to criteria of the US Food and Drug Administration for Enterobacteriaceae [24].

### 3.2. Whole Genome Sequencing and Bioinformatic Analysis

A bacterial genomic DNA purification kit (Tiangen Biochemical Technology, Beijing, China) was used to extract bacterial DNA, which was sequenced using the Illumina and PacBio Sequel sequencing platforms. De novo genome assembly of all isolates in this study was performed using Velvet (Ridom GmbH, Münster, Germany) [25] and annotated using Prokka software [26] and Rapid Annotation using Subsystems Technology (RAST; [27]). The isolate was identified at the species level using Ribosomal Multilocus Sequence Typing [28] and screened for potential antimicrobial resistance genes using ResFinder [29] and for potential virulence-related genes with VFDB [30]. Roary [31] and SEED viewer version 2.0 [32] were used for genome comparison. BLAST [33] and CLC Sequence Viewer 8 were used for comparative genomic analysis. The complete sequence of the AJ6079 has been deposited in GenBank under CP099548 and PubMLST. 

### 3.3. Transmission Electron Microscope (TEM)

The structural characteristics of AJ6079 and colistin-susceptible *A. junii* AOR27 were observed using TEM. The bacterial solution incubated overnight was centrifuged and immobilized in 3% glutaraldehyde for 2.5 h. The samples were observed using a Tecnai Spirit transmission electron microscope (FEI Company, Hillsboro, OR, USA) after treatment in the electron microscope room of the Peking University People’s Hospital (Baita Temple Branch).

### 3.4. Growth Curve Assay

Growth curve assays were performed in quadruplicate for AJ6079 and AOR27 [34]. Bacterial cultures grown overnight in Luria Bertani (LB) broth with rotary agitation at 300 rpm and 37 °C were diluted, individually transferred to 96-well microplates, and incubated. The growth of bacteria was determined by measuring the optical density at 600 nm (OD_600_) every 0.5 h for 30 h. Growth rates were computed by fitting the growth data to a logistic growth curve using GraphPad Prism9 with the equation:

Y = Y^M^ × Y^0^/((Y^M^−Y^0^) × exp(−k × x) + Y^0^),

where Y refers to OD_600_ values and x refers to time points. Y^0^ and Y^M^ represent OD_600_ values at time points initial and maximum population, respectively. k is a constant of the nonlinear fitting logistic growth calculated automatically using the software, representing the growth rate of the isolate.

### 3.5. Statistical Analysis

Statistical analysis of independent samples was performed using the corrected Welch’s *t*-test to determine the differences in the growth curves. GraphPad Prism9 (GraphPad Software Inc., San Diego, CA, USA) was used for data entry and analysis.

## 4. Conclusions

In this study, one clinical *A. junii* isolate, AJ6079, which is resistant to colistin but susceptible to carbapenem, was identified. Through genomic comparative analysis, we found that the colistin-resistant phenotype may be related to multiple mutations in colistin-resistance-related genes *pmrB*, *pmrC*, *lpxA*, and *lpxD*. Growth analysis indicated that the colistin-resistant isolate AJ6079 had a higher fitness cost, restricting the clinical prevalence. Further functional analysis of resistance-related mutations and collection of epidemiological data related to colistin resistance in *A. junii* are required to verify our hypothesis.

## Figures and Tables

**Table 1:** Antibiotics resistance profile of *Acinetobacter junii* AJ6079 and AOR27.

MIC (mg/L)	Imipenem	Meropenem	Colistin	Tigecycline	Ceftazidime	Amikacin	Trimethoprim/ Sulfamethoxazole	Eravacycline	Levofloxacin	Minocycline
AJ6079	≤0.25 (S)	≤0.25 (S)	4 (R)	0.125 (S)	1 (S)	2 (S)	1 (S)	≤0.032 (S)	≤0.125 (S)	≤0.125 (S)
AOR27	≤0.25 (S)	≤0.25 (S)	0.5 (S)	0.125 (S)	0.5 (S)	2 (S)	≤ 0.125 (S)	≤0.032 (S)	≤0.125 (S)	≤0.125 (S)

S, susceptible; R, resistant.

## Data Availability

The data presented in this study are openly available in GenBank at [10.1093/nar/gks1195], reference number [CP099548].

## Applications

Part of this content was published as a conference abstract at the ISAAR conference.
